# Phosphatidylinositol 3-Kinase Mediates Bronchioalveolar Stem Cell Expansion in Mouse Models of Oncogenic *K-ras*-Induced Lung Cancer

**DOI:** 10.1371/journal.pone.0002220

**Published:** 2008-05-21

**Authors:** Yanan Yang, Kentaro Iwanaga, Maria Gabriela Raso, Marie Wislez, Amy E. Hanna, Eric D. Wieder, Jeffrey J. Molldrem, Ignacio I. Wistuba, Garth Powis, Francesco J. Demayo, Carla F. Kim, Jonathan M. Kurie

**Affiliations:** 1 Department of Thoracic/Head and Neck Medical Oncology, University of Texas M. D. Anderson Cancer Center, Houston, Texas, United States of America; 2 Department of Pathology, University of Texas M. D. Anderson Cancer Center, Houston, Texas, United States of America; 3 Service de Pneumologie, Assistance Publique-Hôpitaux de Paris (AP-HP), Hôpital Tenon, UFR Pierre et Marie Curie, Université Paris VI, Paris, France; 4 Department of Stem Cell Transplantation Research, University of Texas M. D. Anderson Cancer Center, Houston, Texas, United States of America; 5 Department of Experimental Therapeutics, University of Texas M. D. Anderson Cancer Center, Houston, Texas, United States of America; 6 Department of Molecular and Cellular Biology, Baylor College of Medicine, Houston, Texas, United States of America; 7 Children's Hospital Stem Cell Program and Department of Genetics, Harvard Medical School and Harvard Stem Cell Institute, Boston, Massachusetts, United States of America; City of Hope Medical Center, United States of America

## Abstract

**Background:**

Non-small cell lung cancer (NSCLC) is the most common cause of cancer-related death in Western countries. Developing more effective NSCLC therapeutics will require the elucidation of the genetic and biochemical bases for this disease. Bronchioalveolar stem cells (BASCs) are a putative cancer stem cell population in mouse models of oncogenic *K-ras*-induced lung adenocarcinoma, an histologic subtype of NSCLC. The signals activated by oncogenic K-ras that mediate BASC expansion have not been fully defined.

**Methodology/Principal Findings:**

We used genetic and pharmacologic approaches to modulate the activity of phosphatidylinositol 3-kinase (PI3K), a key mediator of oncogenic *K-ras,* in two genetic mouse models of lung adenocarcinoma. Oncogenic *K-ras*-induced BASC accumulation and tumor growth were blocked by treatment with a small molecule PI3K inhibitor and enhanced by inactivation of *phosphatase and tensin homologue deleted from chromosome 10*, a negative regulator of PI3K.

**Conclusions/Significance:**

We conclude that PI3K is a critical regulator of BASC expansion, supporting treatment strategies to target PI3K in NSCLC patients.

## Introduction

Non-small cell lung cancer (NSCLC) is the leading cause of cancer-related death in Western countries and its incidence is rising in Asia. Once it has metastasized, there are no curative therapies for NSCLC. A better understanding of the pathogenesis and risk factors for this disease will contribute not only to improvements in early detection and prevention but also to the development of more effective therapies for it.

Approximately 10% of NSCLC specimens carry activating mutations in *K-ras* [1]. The histologic subtype of NSCLC that most frequently has *K-ras* mutations is adenocarcinoma; 30% of adenocarcinomas have these mutations. *K-ras* mutations have also been identified in atypical adenomatous hyperplasia (AAH) lesions, which are thought to precede the development of lung adenocarcinoma [2]. A growing body of evidence indicates that *K-ras* mutations are important in the initiation of lung adenocarcinoma development. Mouse models have been developed that express mutant *K-ras* conditionally, somatically, or inducibly [3]–[7]. These mice develop AAH lesions rapidly and with high penetrance, and a subset of these lesions progress to adenocarcinomas.

A candidate lung cancer progenitor cell (bronchioalveolar stem cell or BASC) has been identified in murine models of *K-ras*-induced lung cancer. BASCs have a specific pattern of stem cell marker expression (Sca-1^pos^ CD34^pos^ CD45^neg^ Pecam^neg^), are located at the terminal bronchi, and have the capacity to self-renew and undergo multi-linage differentiation [8]. These cells may be the same as the variant Clara (or Clara^V^) cells, which are located adjacent to clusters of neuroendocrine bodies in bronchi and bronchioles. Similar to BASCs, Clara^V^ cells are capable of restoring the Clara cell population after naphthalene injury [9]. After intra-nasal installation of adenoviral Cre into Kras^LSL^ mice, which develop lung adenocarcinoma due to activation of a conditional oncogenic *K-ras* allele [5], BASCs rapidly proliferate prior to the development of histologic abnormalities [8]. The BASCs in these mice contain *K-ras* mutations identical to those found in tumors [8]. On the basis of this evidence, BASCs are postulated to be progenitors of lung adenocarcinoma in mice. The biochemical signals that initiate the expansion of this cellular population have not been fully elucidated. This deficiency is important given that the elucidation of these signals might lead to better treatment for NSCLC patients.

Premalignant lesions typically undergo a transient expansion followed by programmed senescence, and only a minority of early lesions progresses to a fully transformed state. In the case of Ras-dependent cancer mouse models, the programmed senescence of premalignant lesions is activated by mitogen-activated protein kinase kinase/extracellular signal-regulated kinase signaling, which inhibits Ras through feed-back pathways involving increased expression of SPROUTY proteins, Ras GTPase activating proteins, and mitogen-activated protein kinase phosphatase-3 [10]. The senescence program in premalignant lesions can be blocked by phosphatidylinositol 3-kinase (PI3K) activation [10], indicating that PI3K is a crucial determinant of the malignant progression of these lesions. In fact, PI3K signaling is constitutively activated in NSCLC cells through inactivating somatic mutations in, or epigenetic silencing of, *phosphatase and tensin homologue deleted from chromosome 10* (*Pten*), a lipid phosphatase that negatively regulates the PI3K signaling cascade [11]–[15].

In this study, we evaluated the importance of PI3K in regulating the expansion of BASCs in two genetic mouse models of oncogenic *K-ras-*induced lung cancer. The oncogenic *K-ras* alleles in these models are activated somatically in one (Kras^LA1^) and conditionally in the other (Kras^LSL^) (5, 7). We found increased numbers of BASCs in these mice relative to those of wild-type littermates. BASC expansion and malignant progression in the lung were attenuated by pharmacologic inhibition of PI3K and enhanced by genetic inactivation of *Pten*. We speculate on the basis of these findings that pharmacologic strategies to inhibit PI3K will be useful in the prevention and treatment of NSCLC.

## Results

### PX-866 inhibits lung tumorigenesis in Kras^LA1^ mice

**Figure 1:**
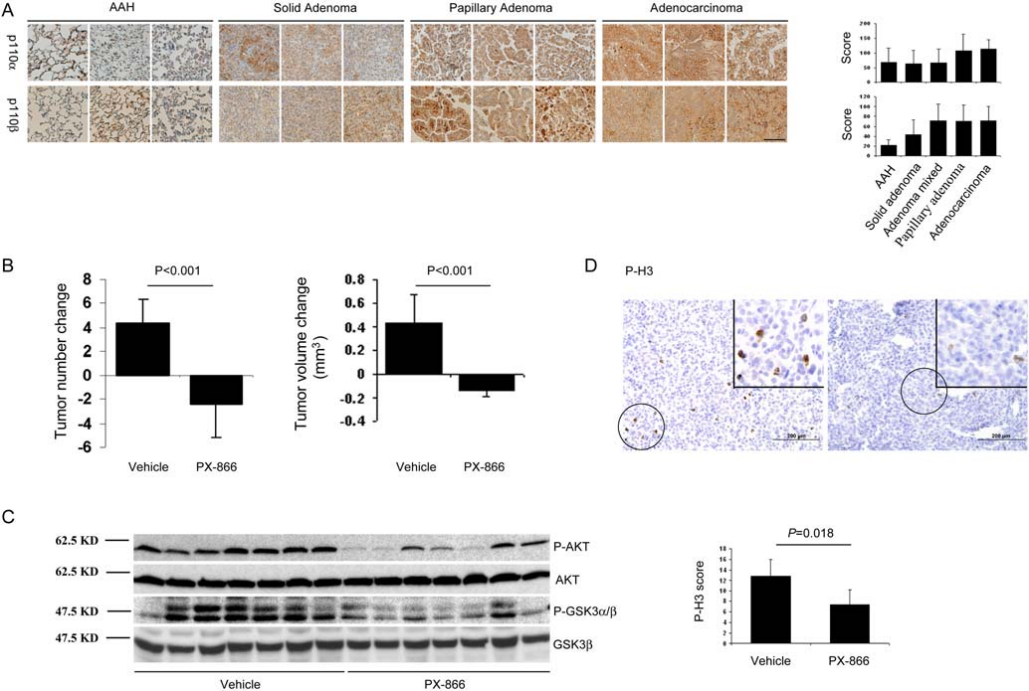
PI3K promotes tumorigenesis in Kras^LA1^ mice. (A) PI3K expression increases with malignant progression. Representative immunohistochemical staining of lesions in the tissue microarray and their quantification in adjacent bar graphs. Calibration bar in lower right panel represents 100 μm. Scores of mixed (solid/papillary) adenomas are included in bar graphs but not in PI3K staining images. (B) PI3K is required for tumor growth. Mean changes in tumor multiplicity and volume determined by micro-computed tomography performed before and after treatment. (* *P*<0.05 compared to vehicle). (C) PX-866 inhibits PI3K in lung tissues. Western blotting of Ser473-phosphorylated AKT (P-AKT) and Ser21/9-phosphorylated GSK3 (P-GSK3α/β) in whole lung lysates from mice (n = 7 in each treatment cohort). Positions of molecular weight markers are indicated on the left. (D) PX-866 decreases tumor cell proliferation. Representative immunohistochemical staining of Ser28-phosphorylated histone H3 (P-H3) in adenomas in the tissue microarray (x 20 magnification, areas with positive cells encircled) and quantification of staining in 16 lesions from vehicle-treated mice and 4 lesions from PX-866-treated mice (bar graph, * *P*<0.05 compared to vehicle). Inset illustrates encircled cells at ×40 magnification. Calibration bars represent 200 μm.

We postulated that PI3K is required for malignant progression in lung cancer and tested this hypothesis by using Kras^LA1^ mice as a model of lung tumorigenesis. Several weeks after birth, Kras^LA1^ mice exhibit multifocal AAH lesions that, by 2–3 months of age, coalesce into solid or papillary adenomas, which enlarge and undergo histologic transformation into adenocarcinomas by 6–8 months [7]. We first evaluated the expression of PI3K (p110α and β isoforms) in lung tissues at different stages of tumorigenesis (AAH, adenoma, or adenocarcinoma). These PI3K isoforms were detected and their abundance increased with malignant progression (AAH lesions *versus* adenocarcinomas: *P* = 0.006 for p110α; *P*<0.001 for p110β) (Figure 1A).

Based on its increased abundance in fully transformed cells, we next sought to determine whether PI3K was required for tumor growth. Treatment of K-ras^LA1^ mice with PX-866, a PI3K inhibitor [16], decreased lung lesion volume and multiplicity (Figure 1B). This was accompanied by reductions in PI3K activity as shown by western blotting of whole-lung lysates for phosphorylated AKT and glycogen synthase kinase-3 (Figure 1C). Tumor cells underwent a reduction in proliferation caused by PX-866 based on intra-lesional phosphorylated histone H3 (Figure 1D) but exhibited no biochemical evidence of apoptosis as shown by western blotting for cleaved caspase-3 and Poly (ADP-ribose) polymerase (data not shown). Thus, treatment with a PI3K antagonist profoundly inhibited the growth of these early lesions.

### BASCs are highly sensitive to PX-866 *in vivo* and *in vitro*

**Figure 2:**
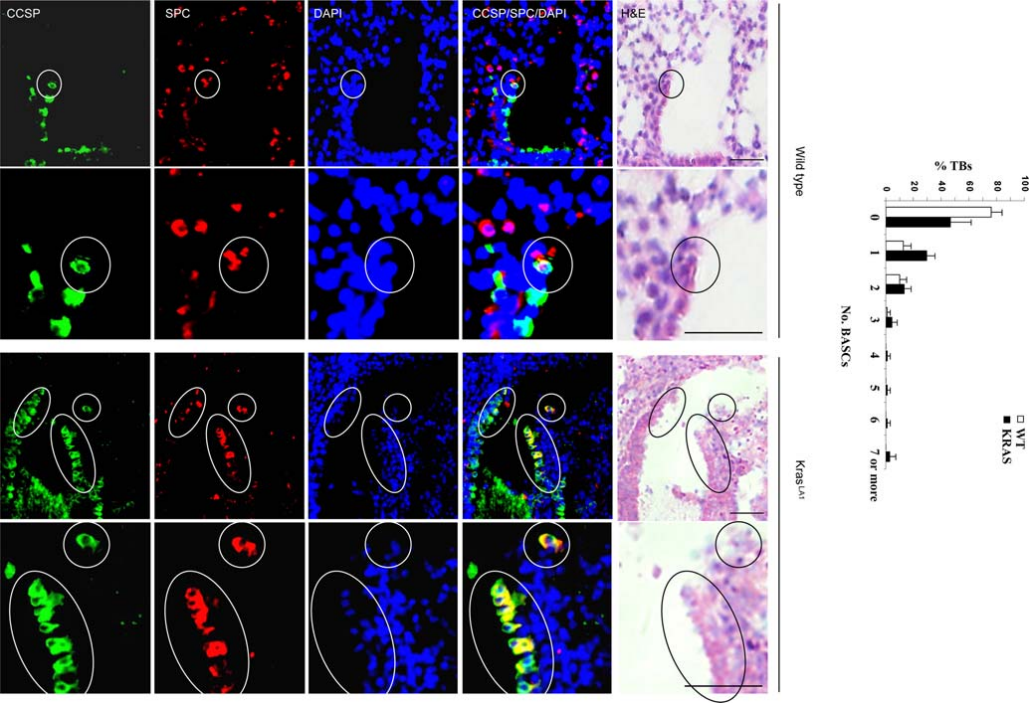
Kras^LA1^ mice have an accumulation of BASCs at terminal bronchi. Immunofluorescent staining to detect cells at terminal bronchi that co-express CCSP and SPC. Encircled BASCs in top panels (x10 magnification) illustrated in lower panels at higher magnification (x40). Bar graph indicates percentages of terminal bronchi with the indicated numbers of BASCs. Calibration bars in images of H&E stained tissues represent 50 μm.

**Figure 3:**
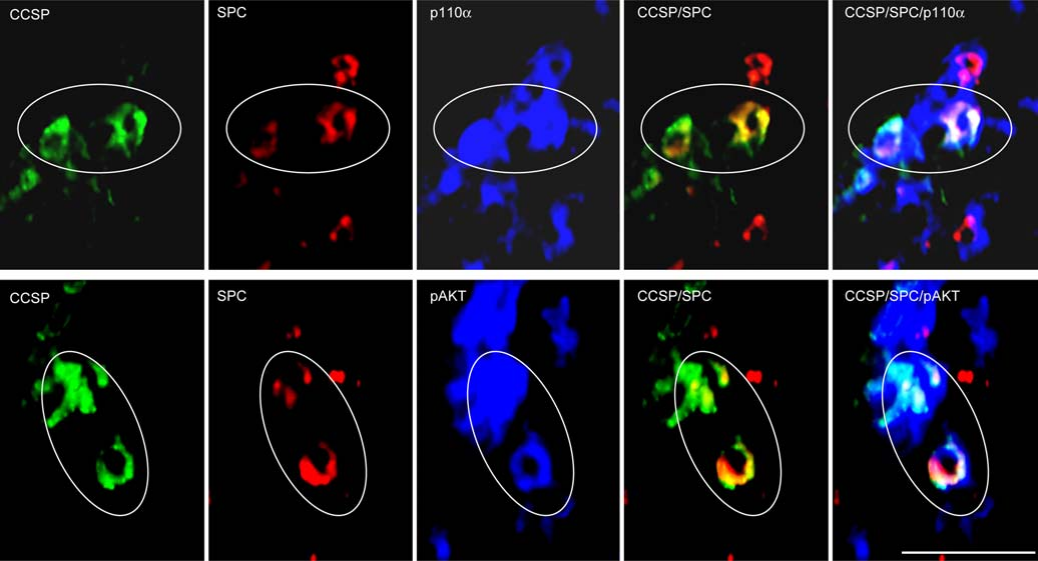
BASCs express p110α and have Ser473-phosphorylated AKT. Immunofluorescent staining of tissue sections to detect triply stained (CCSP/SPC/p110α or pAKT) cells at terminal bronchi. BASCs encircled (x40 magnification). Calibration bar in lower right panel represents 50 μm.

**Figure 4:**
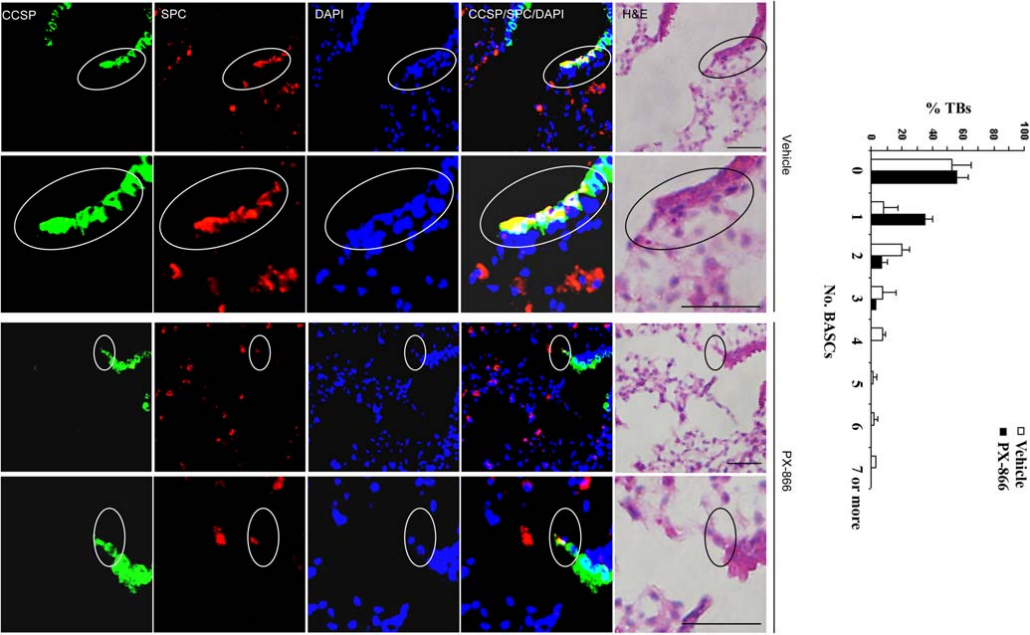
PX-866 treatment decreases BASC numbers in Kras^LA1^ mice. Immunofluorescent staining to detect cells at terminal bronchi that co-express CCSP and SPC. Encircled BASCs in top panels (×10 magnification) illustrated in lower panels at higher magnification (×40). Bar graph indicates percentages of terminal bronchi with the indicated numbers of BASCS. Calibration bars in images of H&E stained tissues represent 50 μm.

We next determined whether the anti-tumor effect of PX-866 in Kras^LA1^ mice was partly due to inhibition of BASC expansion. At the terminal bronchi, a subset of epithelial cells expressed both clara cell specific protein (CCSP) and surfactant protein-C (SPC) (Figure 2), which is a hallmark of BASCs [8]. Quantification of dual-positive cells in wild-type mice (with 83 bronchi) and K-ras^LA1^ mice (with 66 bronchi) revealed that no wild-type mice had more than 3 BASCs per terminal bronchus, whereas a subset of terminal bronchi in K-ras^LA1^ mice had 4 to 7 (*P* = 0.042, wild-type *versus* Kras^LA1^) (Figure 2). Thus, a subset of terminal bronchi in Kras^LA1^ mice had BASC expansion. Furthermore, triple immunofluorescence studies revealed that BASCs expressed p110α and had detectable phosphorylation of AKT (Figure 3), a downstream mediator of PI3K, providing evidence of PI3K activation in these cells. Using tissue sections from the Kras^LA1^ mice treated with PX-866 (with 88 terminal bronchi) or vehicle (with 81 terminal bronchi), we determined the number of BASCs per terminal bronchus in the treatment groups. The vehicle-treated mice had a subset of terminal bronchi with 4 to 7 BASCs per terminal bronchus, whereas none of the PX-866-treated mice had more than three BASCs per terminal bronchus (*P* = 0.025, vehicle *versus* PX-866-treated) (Figure 4).

**Figure 5:**
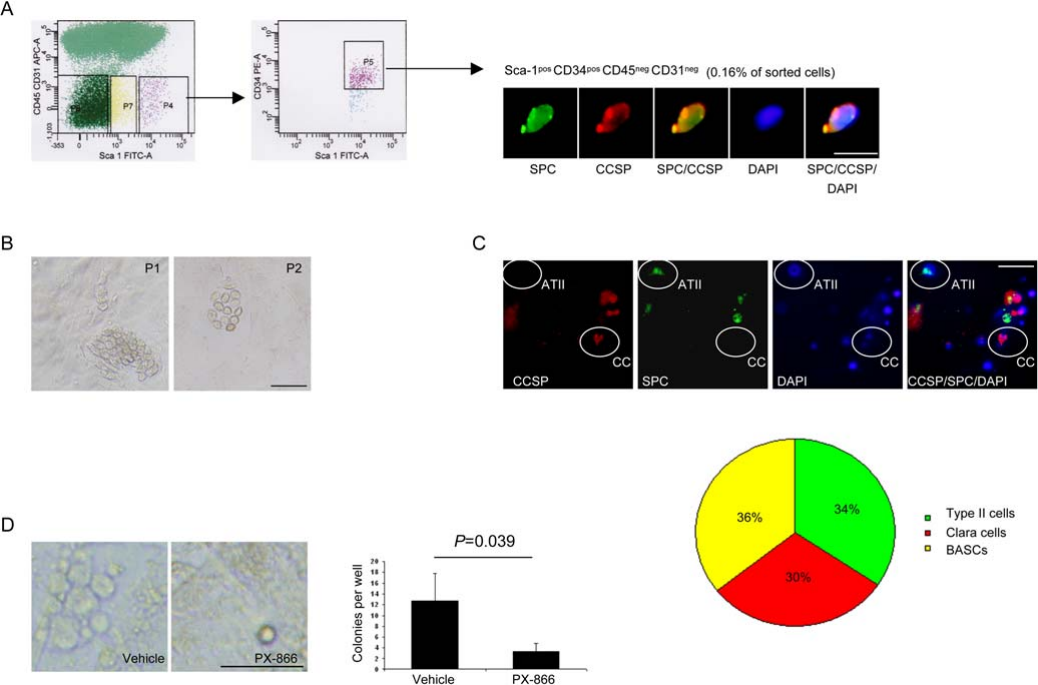
PX-866 inhibits BASC expansion *in vitro.* (A) Sorted cells co-express SPC and CCSP. Kras^LA1^ whole lung tissues were dissociated and sorted to isolate Sca-1^pos^ CD45^neg^ CD31^neg^ CD34^pos^ cells (P4 and P5 in left and middle panels, respectively), which were subjected to immunofluorescent staining (x40 magnification, right panel). Calibration bar in far right panel represents 10 μm. (B) BASCs form colonies when plated on feeder cultures. Photograph of colony (x10 magnification) formed upon initial plating (P1) and passage 2 (P2). Calibration bar in panel on right represents 50 μm. (C) BASCs differentiate when plated in matrigel. Cells were stained after 7 d in matrigel (x10 magnification) and the numbers of cells with features of alveolar type II cells (CCSP^neg^ SPC^pos^), clara cells (CCSP^pos^ SPC^neg^), or BASCs (CCSP^pos^ SPC^pos^) were counted and expressed as the percentages of the total 158 counted cells in a pie chart. Examples of cells with features of ATII cells (ATII) and clara cells (CC) are indicated. Calibration bar in far right panel represents 50 μm. (D) PX-866 inhibits BASC colony formation. Photographs (x10 magnification) and quantification of colonies per well (5 wells per condition) formed after 6 d in the presence or absence of PX-866. Calibration bar in panel on right represents 50 μm.

To determine whether PX-866 had direct effects on Kras^LA1^–derived BASCs, we used primary BASC cultures, which were isolated from the lungs of Kras^LA1^ mice by sorting for cells that were Sca-1^pos^, CD34^pos^, CD45^neg^, and CD31^neg^. These sorted cells co-expressed CCSP and SPC (Figure 5A), formed colonies when plated on feeder cultures (Figure 5B), and exhibited multi-potential differentiation capacity when plated in matrigel (Figure 5C), which are hallmarks of BASCs [8]. PX-866 treatment led to a prominent decrease in BASC colony forming activity (Figure 5D), indicating that PX-866 had a direct effect on BASCs.

### *Pten* deficiency enhances oncogenic *K-ras*-induced BASC accumulation

As another approach to evaluating the role of PI3K in BASC expansion, we inactivated *Pten*, a negative regulator of PI3K, in CCSP-expressing cells. *Pten* was genetically inactivated by using *Pten* ^flox/+^ mice, which have a *Pten* allele that contains *LoxP* sites surrounding the PTEN phosphatase domain encoded by exon 5 [17]. *Pten* was recombined specifically in the lung by interbreeding these mice with CCSP^Cre/+^ mice, which express Cre under the control of the *CCSP* gene [18]. In addition, we evaluated whether *Pten* inactivation cooperated with oncogenic *K-ras* to promote BASC expansion. *Pten*-deficient mice were interbred with K-ras^LSL^ mice, in which the expression of oncogenic *K-ras^G12D^* is controlled by a removable transcription termination STOP element [5]. Using this approach, mice were generated with bronchi that are *Pten*-deficient (Pten^Δ5/Δ5^; CCSP^Cre/+^), express oncogenic *K-ras* (Kras^Lox/+^; CCSP^Cre/+^), have *Pten* deficiency and oncogenic *K-ras* expression (Pten^Δ5/Δ5^; Kras^Lox/+^; CCSP^Cre/+^), or have neither (Pten^flox/flox^; CCSP^+/+^). Mice were sacrificed at 4 weeks or 12 weeks, and their lung tissues were subjected to immunofluorescence studies to quantify BASCs.

**Figure 6:**
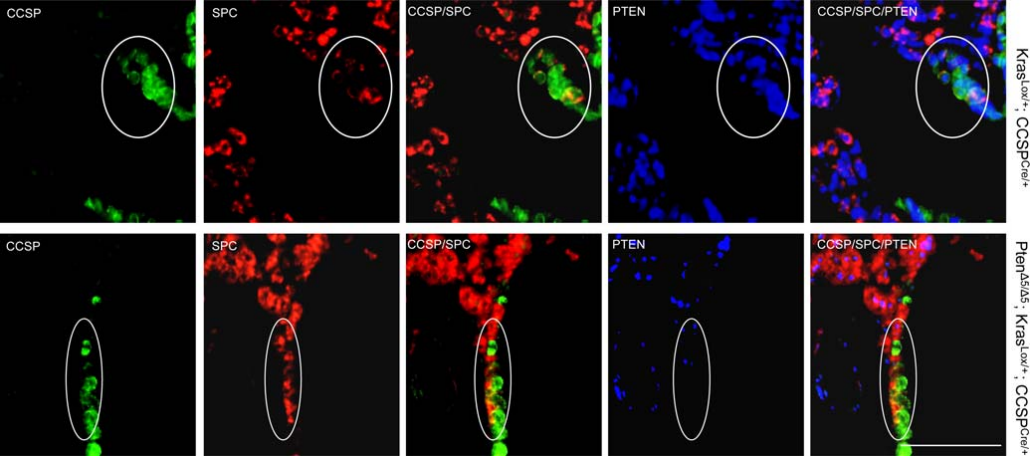
Loss of PTEN expression in BASCs from Pten^Δ5/Δ5^; Kras^Lox/+^; CCSP^Cre/+^ mice. Immunofluorescent staining of tissue sections to detect triply stained (CCSP/SPC/PTEN) cells at terminal bronchi. BASCs (encircled) illustrated at ×10 magnification. Calibration bar in lower right panel represents 100 μm.

**Figure 7:**
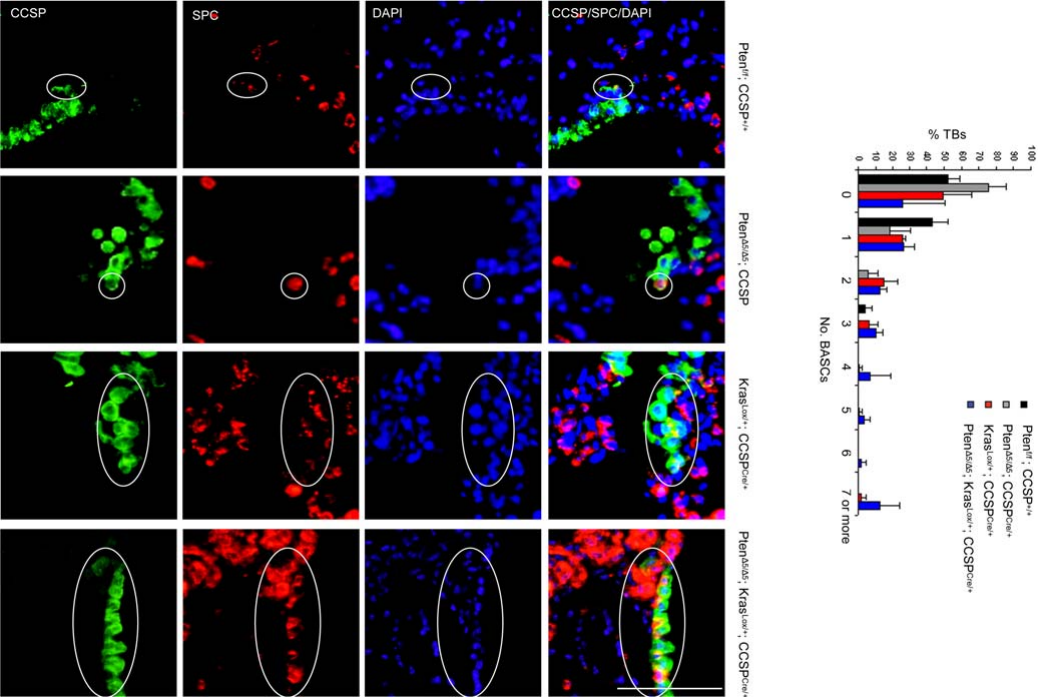
BASC expansion at terminal bronchi of Pten^Δ5/Δ5^; Kras^Lox/+^; CCSP^Cre/+^ mice. Immunofluorescent staining to detect cells at terminal bronchi that co-express CCSP and SPC. BASCs (encircled) illustrated at ×40 magnification. Bar graph indicates percentages of terminal bronchi with the indicated numbers of BASCS. Calibration bar in lower right panel represents 100 μm.

**Figure 8:**
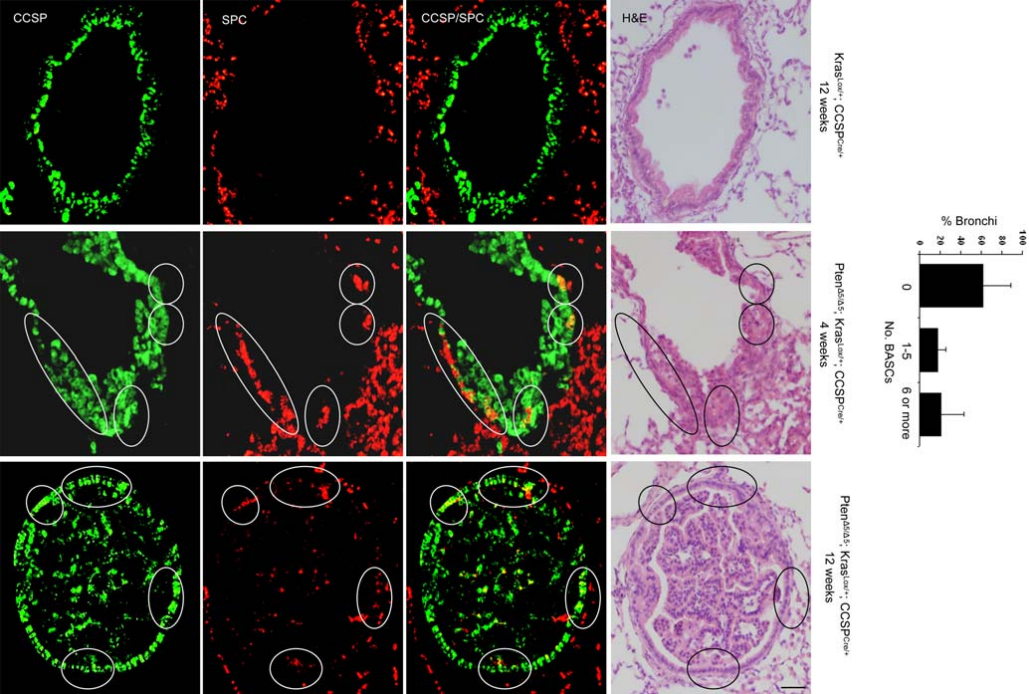
BASCs identified in small bronchi/bronchioles of Pten^Δ5/Δ5^; Kras^Lox/+^; CCSP^Cre/+^ mice but not Kras^Lox/+^; CCSP^Cre/+^ mice. Immunofluorescent staining to detect cells that co-express CCSP and SPC. BASCs (encircled) illustrated at ×10 magnification. Bar graph illustrates percentages of bronchi with the indicated numbers of BASCS. Calibration bar in lower right panel represents 100 μm.

As expected, triple immunofluorescence staining (CCSP/SPC/PTEN) revealed loss of PTEN expression in BASCs from mice that were both *Pten*-deficient and *K-ras*-transformed (Pten^Δ5/Δ5^; Kras^Lox/+^; CCSP^Cre/+^) but not in mice that were only *K-ras*-transformed (Kras^Lox/+^; CCSP^Cre/+^) (Figure 6). Quantification of BASCs per terminal bronchus at 4 weeks revealed that *Pten*-deficiency alone (Pten^Δ5/Δ5^; CCSP^Cre/+^) was not sufficient to change BASC numbers relative to that of control mice (Pten^flox/flox^; CCSP^+/+^) (Figure 7). However, comparison of BASC numbers in Pten^Δ5/Δ5^; Kras^Lox/+^; CCSP^Cre/+^ mice to that of Kras^Lox/+^; CCSP^Cre/+^ mice demonstrated that *Pten* deficiency strikingly enhanced BASC expansion by oncogenic *K-ras* (*P* = 0.006, Pten^Δ5/Δ5^; Kras^Lox/+^; CCSP^Cre/+^ *versus* Kras^Lox/+^; CCSP^Cre/+^) (Figure 7). In addition to these findings at the terminal bronchi, clusters of CCSP^pos^ SPC^pos^ cells were observed in the bronchioles and small bronchi of Pten^Δ5/Δ5^; Kras^Lox/+^; CCSP^Cre/+^ mice; these cell clusters were evident as early as 4 weeks of age and were not present in the bronchi or bronchioles of Kras^Lox/+^; CCSP^Cre/+^ mice (Figure 8). We conclude that *Pten* inactivation cooperated with oncogenic *K-ras* to enhance BASC accumulation.

## Discussion

Here we report that PI3K was required for BASC expansion initiated by oncogenic *K-ras* and, when constitutively activated by *Pten* inactivation, PI3K cooperated with oncogenic *K-ras* in this process. This conclusion was based on findings from experiments that incorporated pharmacologic and genetic approaches to target PI3K in two different genetic mouse models and an *in vitro* model. We have previously reported that *Pten* inactivation cooperates with oncogenic *K-ras* to accelerate lung tumorigenesis, causing lung tumors that are more histologically advanced and more rapidly obstruct bronchial lumina and replace alveolar spaces than those of mice with oncogenic *K-ras* alone [18]. Together, these findings raise the possibility that PI3K promoted lung tumorigenesis through its effects on BASCs. However, conclusive evidence in this regard will require proof that adenocarcinomas arise from BASCs. Transplantation studies to determine whether BASC injections are sufficient to recapitulate lung adenocarcinoma development in mice have thus far not been reported. A stem cell population has been identified in the lungs of mice and in tumors from NSCLC patients that expresses CD133, exhibits stem cell characteristics *in vitro* (forms spheres and can be induced to undergo terminal differentiation), and is tumorigenic in nude mice [19]. Although certain features of that stem cell population are similar to those of BASCs (i.e. response to naphthalene-induced lung injury), their relationship remains to be elucidated.

When initiated during embryogenesis, conditional *Pten* inactivation in the lung leads to hypoxia and perinatal death, indicating that *Pten* expression is required for normal lung development [20]. In this study, Cre expression in CCSP^Cre^ mice began at postnatal day 4, allowing us to evaluate *Pten*'s role in maintaining lung function after birth. Although we can not exclude the possibility that subtle changes in lung function occurred, the *Pten*-deficient mice in this study had no obvious alterations in their lung structure and exhibited no morbidity at up to 12 months of age, suggesting that, after birth, lung tissues can function normally without *Pten*. This apparent resilience of lung tissues to *Pten* deficiency stands in contrast to adult hematopoietic stem cells, which are depleted within 40 days after inactivation of *Pten* [21], indicating that the self-renewal capabilities of hematopoietic stem cells can not be maintained in the absence of PTEN's negative growth regulatory signals. Although the reasons for this difference are not clear, it may be related in part to the relative infrequency with which lung stem cells normally divide [9].

PI3K has been reported to play an important role in tumorigenesis initiated by oncogenic *K-ras*. Kras^LA2^ mice that have a knock-in *PIK3CA* mutation that blocks the interaction of PI3K with K-ras develop far fewer lung tumors than do Kras^LA2^ mice with wild-type *PIK3CA* [22], indicating a key role for PI3K as a downstream mediator of K-ras. Corroborating this point, we found that PX-866 treatment inhibited lung tumor growth in Kras^LA1^ mice. However, based on other findings reported here, the importance of PI3K in *K-ras*-induced lung tumorigenesis may not be restricted to its role as a downstream mediator of K-ras. First, PI3K expression increased as Kras^LA1^ lung tissues underwent malignant progression from AAH to adenocarcinoma, suggesting that PI3K was activated in these histologically advanced lesions in part through K-ras-independent mechanisms. Second, findings reported here and elsewhere [18] indicate that *Pten* inactivation enhanced oncogenic *K-ras*–induced lung tumorigenesis in mice. Extrapolating from these findings, somatic mutations in NSCLC cells involving genes that regulate PI3K-dependent signaling (i.e. *Pten, Egfr*, and *PIK3CA*, among others [ref. 23]) may transform these cells in part by cooperating with oncogenic *K-ras*. The potential importance of secondary somatic mutations in promoting oncogenic *K-ras*-induced lung tumorigenesis is illustrated by the long latencies of adenocarcinomas that arise in mice engineered to express mutant *K-ras*, which develop lung lesions rapidly and with high penetrance, but only a fraction of the lesions progress into adenocarcinomas and require several months to do so [3]–[7].

Findings reported here differed from those reported previously in a different strain of mice that undergo conditional *Pten* deletion induced by SPC-driven Cre expression [20]. In that report, *Pten* inactivation was sufficient to increase BASC numbers and induce lung tumors, which stands in contrast to findings presented here and elsewhere [18] that *Pten* deficiency is not sufficient to increase BASC numbers or induce lung tumors. That previous report also differed with respect to the genetic events that cooperated with *Pten* deficiency. In that study, *Pten* inactivation accelerated lung tumorigenesis induced by urethane, a carcinogen that frequently induces *K-ras* mutations, but only 2 of 6 tumors evaluated had *K-ras* mutations, which leaves open the possibility that urethane cooperates with *Pten* loss by inducing somatic mutations of genes other than *K-ras*. This stands in contrast to our model in which the conditional *K-ras* and *Pten* alleles recombined in the same (CCSP-expressing) cells. Hence, the disparate outcomes of these studies might be related to secondary genetic events in the urethane-treated mice or differences in experimental design, such as the genetic backgrounds of the mice or the specific promoter elements driving Cre expression.

## Methods

### Reagents

Antibodies purchased for these studies include rabbit anti-p110α (sc-7174), p110β (sc-7175), SPC (sc-13979), goat anti-CC10 (sc-9772) and PTEN (sc-6818) (Santa Cruz Biotechnology, Santa Cruz, CA), rabbit anti-total AKT (9272), Ser473-phosphorylated AKT (9271), Ser21/9-phosphorylated GSK3α/β (9331), total GSK3 (9332), cleaved caspase-3 (9661), Poly (ADP-ribose) polymerase (9542), phosphorylated-histone H3 (9701), Ser28-phosphorylated histone H3 (9713P) (Cell Signaling Technologies, Danvers, MA), rabbit anti-SPC (WRAB-SPC, Seven Hills Bioreagents, Cincinnati, OH), rabbit anti-CCSP (07-623, Upstate Biotechnologies, Lake Placid, NY), FITC-conjugated anti-Sca-1 (557405), biotin-conjugated anti-CD45 (553771) biotin-conjugated-CD31 (558737), PE-conjugated anti-CD34 (12-0341-33, eBioscience), anti-rabbit IgG Alexa Flour 488 (A11008), and anti-goat IgG Alexa Flour 594 (A11058) (Invitrogen, Carlsbad, CA). Other purchased reagents include streptavidin-APC (554067, BD Pharmigen), TSA kits 22 (T20932), 25 (T20935), and 27(T20937), Avidin/Biotin blocking kit (00-4303) (Invitrogen), Dispase (354235, BD Biosciences), collagenase/dispase (10269638001, Roche Biosciences), and 7-aminoactinomycin D (A1310, Invitrogen).

### Mouse studies

To test whether PI3K promotes tumor development, 9 K-ras^LA1^ mice were treated for 4 weeks with 2 mg/kg/d intra-gastric PX-866, a small molecule inhibitor of PI3K [12], and 12 were given vehicle beginning at 4 months, an age at which lung lesions (AAH and adenomas) are measurable by micro-computed tomography scans. Mice were subjected to these scans at the beginning and completion of treatment to count lesion numbers and evaluate changes in lesion volumes over time as previously reported [24].

Prior to their initiation, all mouse studies were submitted to and approved by the Institutional Animal Care and Use Committee at the University of Texas–M. D. Anderson Cancer Center. Mice received standards of care and were euthanized according to the standards set forth by the IACUC. Mice and cells and were obtained through collaborations with Dr. Tyler Jacks, MIT (Kras^LA1^ mice, and Kras^LSL^ mice), Dr. Hong Wu, UCLA (Pten^flox^ mice), and Dr. Francesco Demayo, Baylor College of Medicine (CCSP^Cre^ mice).

### Tissue microarrays and immunohistochemical analyses

We performed immunohistochemical analysis of PI3K on a tissue microarray containing AAH (n = 17), solid adenomas (n = 31), mixed solid/papillary adenomas (n = 36), papillary adenomas (n = 45), and adenocarcinomas (n = 14) to determine whether the levels of PI3K changed during malignant progression. We analyzed the expression of PI3K isoforms (p110α and β).

For immunohistochemical staining, 4 μm paraffin-embedded sections were baked at 60°C for 30 min and then cooled to ambient temperature. Sections were sequentially incubated in Xylene (5 min twice), 100% ethyl alcohol (5 min twice), 95% ethyl alcohol (5 min twice), and 80% ethyl alcohol (5 min). After washing with water, the sections were antigen-retrieved using citrate buffer (pH 6.0, DAKO) in a steamer for 20 min and cooled to ambient temperature. Sections were then washed with TBS-T and quenched with 3% hydrogen peroxide in TBS for 10 min, blocked for Avidin/Biotin activity, and incubated with primary antibody as follows: For p110α and p110β staining, sections were blocked with 5% goat serum for 1 h, incubated with primary antibodies for 1 h at ambient temperature, and then washed with TBS-T; for Ser28-phosphorylated histone H3 staining, sections were blocked with 10% goat serum at 4°C overnight, incubated with primary antibody for 30 min at ambient temperature, and washed with TBS-T. The staining was developed using DAB substrate system. Negative controls included omission of the primary antibody and pre-incubation of primary antibody with blocking peptide. Staining was quantified by one investigator (M.G.R.) blinded as to treatment group. Intra-lesional expression was scored based on staining intensity and extension as described previously [24].

### Detection of BASCs in lung tissues

Antigens were retrieved from mouse lung tissues with 0.01 mol/L citrate buffer (pH 6; DakoCytomation, Glostrup, Denmark) for 25 min in a steamer. The slides were quenched in 1.5% hydrogen peroxide in Tris-buffered saline for 10 minutes and blocked in DAKO serum-free protein block (Dako) for 1 h at ambient temperature. The slides were then incubated with anti-SPC (sc-7705, Santa Cruz Biotechnology; WRAB-SPC, Seven Hills Bioreagents) and anti-CCSP (07-623, Upstate Biochemicals) antibodies at 4°C overnight. The immunofluorescence was developed using TSA kits 25 and 22 (Invitrogen) on the basis of the manufacturer's instructions. We used blocking peptides and omitted the primary antibodies as negative controls to determine the specificity of the immunostaining results. Immunofluorescence was visualized using a microscope with a reflected fluorescence system (Olympus Model IX71SIF-2). Color acquisition and background fluorescence were optimized by using the DPController and DPManager software (Olympus).

### BASC isolation and culture

The mice were killed after 12 weeks, and the lungs were perfused with 10 mL of Hank's balanced salt solution through the right ventricle until they were cleared of blood. The tracheas were injected with Dispase (undiluted liquid, BD Biosciences) followed by 0.5–1 mL 1% LMP agarose in water. The lungs were placed on ice, chopped into pieces, incubated with 0.001% DNAse (Sigma D-4527) and 2 mg/mL of collagenase/dispase (100 mg/mL, Roche) in phosphate-buffered saline at 37° C for 45 min, filtered (100 μm followed by 40 μm pore-sized filter), and centrifuged. The resulting pellets were resuspended in 2 mL of PF10 (10% fetal bovine serum in phosphate-buffered saline), which typically yields 1–2 million total cells per mouse. The red cells were lysed. BASCs were isolated by sorting of the Sca-1^pos^ CD45^neg^ Pecam^neg^ CD34^pos^ cell population on a three-laser 13-color FACS Aria fluorescence-activated cell sorter (BD Biosciences) that is capable of sorting four sub-populations at a low pressure with a maximum rate of 20 million cells/hour. BASCs represented approximately 0.1% of the total sorted cell population.

The uniformity or purity of the sorted cells was not routinely checked because the cells of interest would have been consumed in the process. That being said, the cells were sort-purified to the highest possible purity by gating on size (by forward and side scatter), excluding doublets (by forward scatter width *versus* area and side scatter width *versus* area), and isolating cells with the markers of interest. In addition, the uniformity of the sorted cell populations used in these assays was reflected by the reproducibility of our findings; the differentiation and colony formation assays were performed each time using cells derived from three different mice, which yielded similar results.

For colony formation assays, the sorted cells were plated in DMEM/HEPES/10% FBS/Pen-Strep/L-glutamine on 96-well plates with feeder cells (irradiated DR4 mouse embryo fibroblasts, SCRC-1045.1, American Type Culture Collection, Manassas, VA) to maintain the undifferentiated state (1000 cells per well). PX-866 was added to the cultures the day following plating. For differentiation assays, BASCs were plated for 7 d on 96-well plates (10,000 cells per well) coated with 100 μL growth factor-reduced Matrigel (356231, BD Biosciences) and subjected to immunofluorescent staining for SPC and CCSP as described [8]. The yield of BASCs (Sca-1^pos^ CD45^neg^ Pecam^neg^ CD34^pos^) is typically 0.1% of sorted cells from the lung.
